# Development of TAP, a non-invasive test for qualitative and quantitative measurements of biomarkers from the skin surface

**DOI:** 10.1186/2050-7771-2-20

**Published:** 2014-11-13

**Authors:** Kadri Orro, Olga Smirnova, Jelena Arshavskaja, Kristiina Salk, Anne Meikas, Susan Pihelgas, Reet Rumvolt, Külli Kingo, Aram Kazarjan, Toomas Neuman, Pieter Spee

**Affiliations:** FibroTx LLC, Mäealuse 4, 12918 Tallinn, Estonia; Dermatology Clinic, Tartu University Hospital, Raja 31, 50407 Tartu, Estonia; PS! Pharmaconsult, Møllemoseparken 44, 3450 Allerød, Denmark

**Keywords:** Biomarker, Dermatology, Skin care, Diagnostics, Transdermal analyses patch, Interleukin 1α, Interleukin 1RA, CXCL-1, CXCL-2, β-defensin-1

## Abstract

**Background:**

The skin proteome contains valuable information on skin condition, but also on how skin may evolve in time and may respond to treatments. Despite the potential of measuring regulatory-, effector- and structural proteins in the skin for biomarker applications in clinical dermatology and skin care, convenient diagnostic tools are lacking. The aim of the present study was to develop a highly versatile and non-invasive diagnostic tool for multiplex measurements of protein biomarkers from the surface of skin.

**Results:**

The Transdermal Analyses Patch (TAP) is a novel molecular diagnostic tool that has been developed to capture biomarkers directly from skin, which are quantitatively analyzed in spot-ELISA assays. Optimisation of protocols for TAP production and biomarker analyses makes TAP measurements highly specific and reproducible. In measurements of interleukin-1α (IL-1α), IL-1 receptor antagonist (IL-1RA) and human β-defensin (hBD-1) from healthy skin, TAP appears far more sensitive than skin lavage-based methods using ELISA. No side-effects were observed using TAP on human skin.

**Conclusion:**

TAP is a practical and valuable new skin diagnostic tool for measuring protein-based biomarkers from skin, which is convenient to use for operators, with minimal burden for patients.

## Background

The human skin is the largest organ of the integumentary system, consisting of multiple layers of ectodermal tissue that protects the underlying muscles, ligaments, bones and internal organs of the body. Cells that reside in the skin, such as keratinocytes, fibroblasts, melanocytes, Merkel cells and Langerhans cells, or immune cells that migrate in and out of the skin, are highly adaptive to the constantly changing outer and inner milieus of the body to prevent e.g. physical damage, temperature changes, dehydration and pathogen invasion [1]. To ensure the integrity of skin, skin cells secrete large amounts of structural, effector and regulatory molecules, including scleroproteins (e.g. collagen type I and IV, keratin) [2–6], cytokines (e.g. IL-1α, IL-1β, IL-1RA, TNF-α and IFN-γ) [7–12], chemokines (e.g. CCL-1) [13, 14], angiogenesis regulators (e.g. VEGF, thrombospondin-1 and -2) [15–17] and anti-microbial peptides (e.g. β-Defensin-1 (hBD-1) and -2 (hBD-2)) [17, 18].

The integrity of skin is constantly threatened by external factors (e.g. mechanical disruption, chemical influences, extensive sun exposure, bacterial infections), but also from ‘within’, for instance due to hormonal changes causing skin ageing or the impact of chronic inflammation (e.g. psoriasis, atopic dermatitis and systemic lupus erythematosus (SLE)). Skin condition is often monitored visually. In dermatology, for instance, clinical scores for psoriasis (Psoriasis Area Severity Index score (PASI) score) and atopic dermatitis (Scoring Atopic Dermatitis (SCORAD) score) are largely based on visual assessments of clinical parameters, such as skin redness, thickness, swelling, crustiness and scaling [19–21]. Alternatively, the condition of skin may be monitored by ultrasound, e.g. to determine the thickness and density of skin layers (epidermis, sub-epidermal low-echogenic band (SLEB) and dermis) [22, 23]. Despite the obvious value of these methods for monitoring skin conditions at any given time, they have little predictive value for how the condition of skin evolves over time, or whether skin will respond to treatment.

Methods that may fulfill such needs are biomarker assessments. Biomarkers are defined as a characteristic that is objectively measured and evaluated as an indicator of normal biological processes, pathogenic processes, or pharmacological responses to therapeutic intervention [24]. Biomarkers have been classified as: 1) Type 0 markers, which correlate longitudinally with the natural history of the disease, 2) Type I markers, which capture the effects of an intervention in accordance with the mechanism of action of the drug, and 3) Type II markers, which are surrogate endpoints for changes that predict clinical benefits [25].

Biomarkers are of particular interest due to their predictive value. For instance, presence of antibodies reactive with cyclic-citrulinated peptide (CCP) in the blood is a fairly accurate predictor of development of rheumatoid arthritis, preceding clinical signs with months to even years. Several studies have identified Type 0 biomarkers for pathological skin conditions such as psoriasis and atopic dermatitis, including biomarkers for keratinocyte activity (e.g. presence of K16) and inflammatory response (e.g. up-regulation of IL-8 and TNF-α) [26–32]. Type II biomarkers have been identified for various psoriasis and atopic dermatitis therapies, including biomarkers representing Th17 pathway [33–35], monocyte activity (e.g. TNF-α) [31], lymphocyte activity (Granzyme B) and type I interferon pathways [16–24]. Of particular interest is a study by Malaviya *et al*., showing that the amount of cleaved Caspase-3-positive cells, accurately predicts response to Enbrel, months before response to therapy can be assessed based on clinical symptoms [25].

Biomarkers are usually biological molecules, such as mRNA or proteins, or metabolites, derived from tissues or body fluids (e.g. blood) via invasive methods. Recently, Portugal-Cohen has published a non-invasive method in which a limited number of soluble biomarkers could be assessed in skin-lavage from lesional skin of a limited number of psoriasis patients and renal failure [7, 36, 37]. A clear increased concentration of IL-1α, TNF-α and IL-6 was found in skin lavage from lesion sites of psoriasis patients, in comparison with skin lavage from non-lesional sites, or from skin of healthy individuals [7]. Similarly, tape stripping of skin, in combination with proteomic analyses of ‘captured’ proteins, has shown increased presence of inflammatory cytokines on inflamed or irritated skin [38–40].

Here we describe the step-by-step development of TAP. The aim of this development was to generate a non-invasive diagnostic tool for skin biomarker measurements, with a sensitivity and dynamic range, as well as convenience of use, which allows biomarker measurements from different skin conditions. To minimize variation between measurements, the core of TAP, consisting of an antibody micro-array that allows multiplex analyses of selected biomarkers, was optimized for optimal and reproducible capturing of analytes from skin. Using IL-1α, IL-1RA, CXCL-1/2 and hBD-1 as model biomarkers, protocols have been developed for qualitative and quantitative analyses of biomarkers from skin using spot-ELISA directly on TAP.

## Results

### Design of the transdermal analyses patch

**Figure 1:**
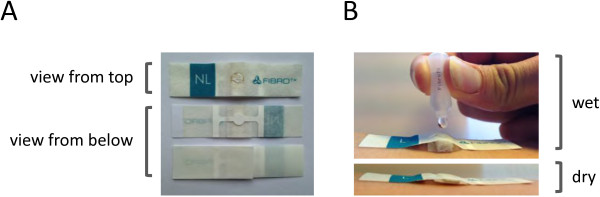
**The Transdermal Analyses Patch (TAP).** Panel **A**: TAP consists of a plaster with a nitrocellulose core that contains the capture antibody micro-array, which is clearly visible in the middle. Panel **B**: In the lower photo, TAP has been fixed to skin. In the upper photo, it is clearly visible that the layer in between the micro-array and the adhesive layer expands upon contact with fluid. This layer serves as a reservoir for the buffer used for biomarker extraction from skin, as well as to ensure close contact between capture antibody micro-array and skin during extraction.

For the selective capturing of soluble proteins directly from skin, we have developed a so-called Transdermal Analyses Patch (TAP), consisting of a micro-array, which is supported by a dermal adhesive plaster for easy fixture to skin (see Figure 1A). TAP micro-arrays contain a panel of capturing antibodies, of which each antibody variant is printed, in multitude, as discrete spots on nitrocellulose by non-contact dispensing. In addition, each TAP micro-array contains a multitude of spots each printed with positive controls (IgG) or negative controls (irrelevant IgG of printer buffer) to determine the specificity of biomarker measurements. In between the antibody micro-array and the plaster, a layer is positioned that serves as a fluid reservoir for the buffer needed for protein capturing from skin. In addition, this expandable layer serves as a pressure pad to ensure close contact of the micro-array to the skin (see Figure 1B). Captured proteins are analyzed, both qualitatively and quantitatively, on the antibody micro-array using spot-ELISA.

### Generation of capture antibody micro-arrays

**Figure 2:**
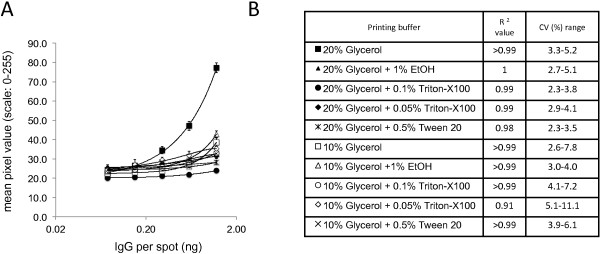
**Effects of different printing buffers on the detection of human IgG printed on nitrocellulose.** Various amounts of human IgG (1.2, 0.6, 0.3, 0.15 and 0.075 ng /spot) were printed on Whatman Protran BA-85 (0.45 μm) membrane using various printing buffers. Printed IgG was visualised in spot-ELISA and signals quantified by determining the pixel intensities of digitized spots. Panel **A**: Each line on graph represents the analyses results of printed IgG in different printing buffer consisting of PBS + 10% glycerol supplemented with either 0.1% or 0.05% Triton X-100, 0.5% Tween-20, 1% ethanol or nothing, or printing buffer consisting of PBS + 20% glycerol, supplemented with either 0.1% or 0.05% Triton X-100, 0.5% Tween-20, 1% ethanol or nothing (see Panel **A** for details). Each data point consists of measurements of five spots on five different strips (N = 25 per data point). X-axis: human IgG amount per spot. Y-axis: Staining intensity defined as the mean pixel intensity measured on a 0–255 grey scale. Panel **B**: R^2^ and CV (Coefficient of variation) value presented for each tested printing buffer.

**Figure 3:**
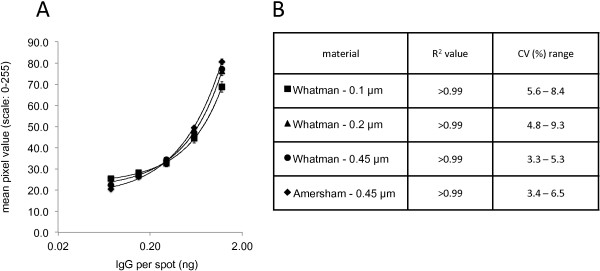
**Effects of different nitrocellulose materials on the detection of printed human IgG.** Panel **A:** Various amounts of human IgG (1.2, 0.6, 0.3, 0.15 and 0.075 ng /spot) were printed on Whatman Protran BA-85 (0.1, 0.2 or 0.45 μm porosity) or Amersham Hybond-C (0.45 μm porosity) membrane using PBS + 20% glycerol as printing buffer. Printed IgG was visualised in spot-ELISA and signals quantified by determining the pixel intensities of digitized spots. Each line on graph represents the analyses results of IgG printed on a specific nitrocellulose (see Panel **A** for details). Each data point consists of measurements of five spots on five different strips (N = 25 per data point). X-axis: human IgG amount per spot. Y-axis: Staining intensity defined as the mean pixel intensity measured on a 0–255 grey scale. Panel **B**: R^2^ and CV% (Coefficient of variation) range for each tested nitrocellulose membrane type.

Accurate TAP biomarker measurements require micro-arrays that stably retain capturing antibodies during analyses and that display minimal variation between individual spots of printed capture antibodies. Therefore, different printing solutions were tested for the generation of stable TAP micro-arrays with minimal variation between printed spots. For this, nitrocellulose strips of Whatman Protran BA 85 nitrocellulose (0.45 μm porosity) were printed with five different amounts (1.2, 0.6, 0.3, 0.15 and 0.075 ng/spot) of human IgG, in five fold, dissolved in PBS formulations containing either 10% or 20% glycerol, that were supplemented or not with various concentrations of either ethanol, Triton-X100 or Tween-20 (see Figure 2 for details). Printed IgG was visualised by spot-ELISA, using HRP-conjugated secondary antibodies specific for the printed IgG. Printed IgG was quantitatively analyzed by determining the pixel intensities of digitised images of the visualised IgG and differences in spot intensities of the spots were measured for each amount of IgG printed with the different printing buffers tested. Regression analyses revealed very strong correlations between spot intensities and amounts of printed proteins for all but one of printing buffers tested (R^2^ ≥ 0.98 for all buffers tested) (see Figure 2). Also, only minor variation between individual spots was observed for all printing buffers tested. CV values did not exceed 10% for any but two of the combinations of printing buffers and IgG amounts tested (see Figure 2). Nonetheless, substantial differences in signal strengths were observed between printing buffers used and how well dispensed capture antibodies were retained on the membrane during the assay. Micro-arrays printed with IgG dissolved in PBS + 20% glycerol without any further additives yielded the highest signal strength observed for the different printing buffers and was therefore chosen for further TAP micro-array development. In a similar fashion, different nitrocellulose membranes were tested, to identify the optimal printing material for the TAP capture antibody micro-arrays. Quantitative analyses of different amounts of IgG printed either on Whatman Protran BA 85 nitrocellulose, with porosities of either 0.1, 0.2 or 0.45 μm, or Amersham Hybond C-Extra, with 0.45 μm porosity, revealed only minor differences in amounts of printed IgG in different spots during analyses (see Figure 3). Very strong correlations were observed between spot intensities and amounts of printed proteins for all materials tested (R^2^ > 0.99). Nonetheless, micro-arrays printed on Whatman Protran BA 85 nitrocellulose with 0.45 μm porosity yielded the lowest CV values (3.3-5.3%) for the different IgG amounts tested, indicating the least variability between individually printed spots, and therefore this material was chosen for further TAP development.

### Optimisation of the protein capture and detection protocol using capture antibody micro-arrays

**Figure 4:**
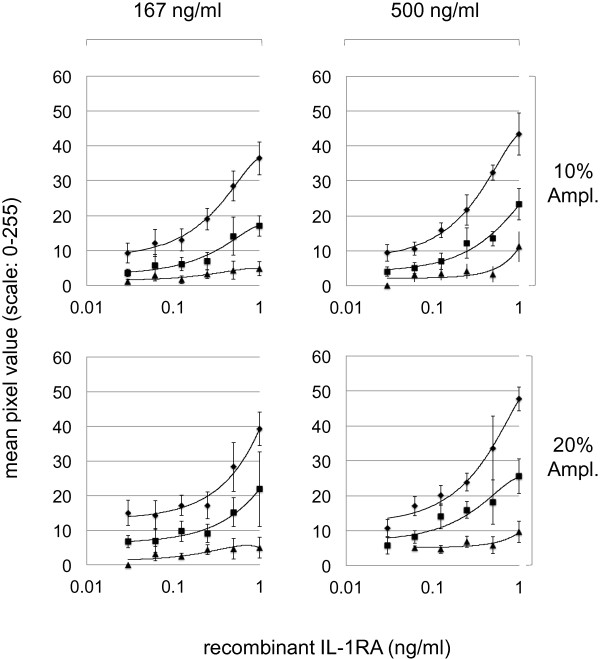
**Measurements of recombinant IL-1RA.** Measurements of recombinant IL-1RA, using 0.75 (triangles), 1.5 (squares) and 2.25 (diamonds) nanogram of anti-IL-1RA capturing antibody printed on nitrocellulose membranes, and a combination of either 500 or 166.67 ng/ml anti-IL-1RA detection antibody and 10% or 20% amplification reagents in spot-ELISA. Each data point consists of 10 measurements (N = 10). Each line presents an average mean of measurements obtained from duplicate. X-axis: Recombinant IL-1RA in ng/ml. Y-axis: Staining intensity defined as the mean pixel intensity measured on a 0–255 grey scale.

Multiplex TAP protein measurements from skin require optimal analytic conditions to secure specific and sensitive measurements as well as a dynamic range that allows protein measurements from different skin conditions. IL-1α, IL-1RA, the combination of CXCL-1 and -2 (referred to as CXCL-1/2) and hBD-1 were chosen as model skin analytes based on literature reports describing that these proteins are expressed in healthy skin, and/or that these proteins have altered expression patterns in lesional (e.g. due to inflammation) or damaged (e.g. due to UV exposure) skin [7, 10–12, 18, 22, 23, 40–43]. For TAP micro-array development, Whatman Protran BA 85 nitrocellulose (0.45 μm porosity) strips were printed with three different amounts of capturing IL-1α, IL-1RA, CXCL-1/2 or hBD-1 antibodies (see Methods for details). Strips were subsequently incubated with different concentrations of recombinant IL-1α, IL-1RA, CXCL-2 or hBD-1. Following washing, strips were incubated with two different concentrations of anti-IL-1α, IL-1RA, CXCL-1/2 and hBD-1 detection antibodies and bound IL-1α, IL-1RA, CXCL-1/2 and hBD-1 were subsequently visualised using two different concentrations of amplification solution and quantitatively analyzed as described. As shown in Figure 4, exemplified by the data obtained for IL-1RA, the major variable that influenced the sensitivity as well as the dynamic range of recombinant IL-1RA measurements was the amount of capture antibody printed on the nitrocellulose strips. The highest amount of anti-IL-1RA used, 2.25 ng per printed spot, yielded the highest sensitivity, signal strength and dynamic range. The highest amount of anti-IL-1RA capture antibodies used, in combination with 10-fold diluted amplification reagents yielded the highest curve fit (R^2^ > 0.99). For the detection of IL-1RA from skin, 2.25 ng capture anti-IL-1RA per spot was chosen for printing TAP micro-arrays, in combination with a spot-ELISA protocol using 166.67 ng/ml detection anti-IL-1RA and a 1/10 fold dilution of amplification reagent. Optimal use of reagents for detection of IL-1α, CXCL-1/2 and hBD-1 were determined in a similar fashion.

To test TAP protein measurements from skin, micro-arrays with anti-IL-1α, -IL-1RA, -CXCL-1/2 and -hBD-1 capturing antibodies were produced that were extensively tested for cross-reactivity of reagents. No cross-reactivities of reagents were observed (data not shown), indicating that TAP measurements of IL-1α, IL-1RA, CXCL-1/2 and hBD-1 from skin should be considered specific.

### Protein measurements from skin using TAP

**Figure 5:**
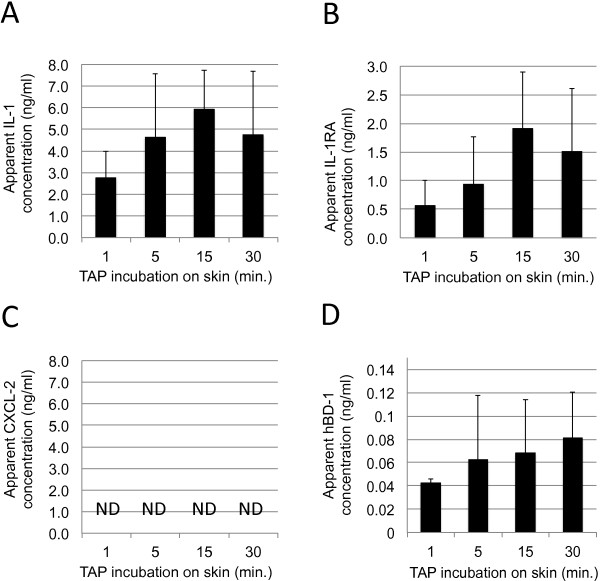
**Measurements of IL-1α, IL-1RA, CXCL-1/2 and hBD-1 from healthy skin.** TAP’s containing capture antibody micro-arrays coated with anti-IL-1α, -IL-1RA, -CXCL-1/2 and -hBD-1 capture antibodies were incubated on skin for 1, 5, 15 or 30 minutes. IL-1α, IL-1RA, CXCL-1/2 and hBD-1 captured from skin were analyzed in spot-ELISA and signals were quantified by determining the pixel intensities of digitized spots. Signal intensities were compared to signals obtained using fixed concentrations of recombinant IL-1α, IL-1RA, CXCL-1/2 and hBD-1 captured in solution. In the graph, the apparent average concentration of IL-1α (Panel **A**), IL-1RA (Panel **B**) CXCL-1/2 (Panel **C**) and hBD-1 (Panel **D**) on skin of three persons has been plotted against incubation time on skin. Each data point in the graphs represents results of three spots analyzed on 6 TAP’s (N = 18). Y-axis: Apparent concentration of IL-1α, IL-1RA, CXCL-1/2 or hBD-1 on skin in ng/ml. X-axis: Incubation time on skin in minutes. Error bars on graph present standard deviation of analyzed data points (N = 18).

TAP containing anti-IL-1α, -IL-1RA, -CXCL-1/2 and -hBD-1 capturing antibody micro-arrays were applied to normal, non-sun exposed skin on the inside of the lower arm of healthy volunteers (N = 3) for 1, 5, 15 or 30 minutes. Captured IL-1α, IL-1RA, CXCL-1/2 and hBD-1 were subsequently visualised using anti-IL-1α, -IL-1RA, -CXCL-1/2 and -hBD-1 detection antibodies in spot-ELISA, and quantitatively analyzed, as described. As shown in Figure 5, IL-1α, IL-1RA and hBD-1 could be efficiently detected on normal skin using TAP, whereas CXCL-1/2 was not detectable. Maximal signal strength was obtained following 15 minutes incubation on skin, however, no statistically significant differences were observed between 5, 15 and 30 minutes. An incubation time of 20–30 minutes was used in following experiments, to minimize variability in measurements due to differences in incubation times on skin.

### Reproducibility of TAP biomarker measurements from skin

**Figure 6:**
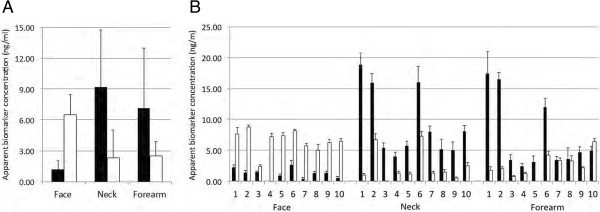
**Measurements of IL-1** **α and IL-1RA from three different skin regions on healthy volunteers on five consecutive days.** TAP’s containing capture antibody micro-arrays coated with anti-IL-1α and -IL-1RA capture antibodies were incubated on skin of the inner side of the lower arm (‘Forearm’), cheek (‘Face’) or collar bone (‘Neck’) regions of ten healthy volunteers for 20 minutes. The capturing procedure was repeated on the four following days on exact the same regions, at around the same time-point of day. IL-1α and IL-1RA captured from skin were analyzed in spot-ELISA and signals were quantified by determining the pixel intensities of digitized spots. Signal intensities were compared to signals obtained using fixed concentrations of recombinant IL-1α and IL-1RA, captured in solution. In graph **A**, the apparent average concentrations of IL-1α black bars and IL-1RA (white bars) have been plotted for the three different body regions. In graph B, the apparent average concentrations of IL-1α black bars and IL-1RA (white bars) have been plotted for the three different body regions for each of the ten persons (1–10 in graphs) analysed. Y-axis: Apparent concentration of IL-1α and IL-1RA on skin in ng/ml. X-axis: Individual participants labeled 1–10. Error bars in graph A represent the standard deviations from average of combined measurements in the 10 healthy volunteers. Error bars in graph **B**, represent the standard deviations from the average of measurements from five different days for each of the healthy volunteers.

To measure the reproducibility of TAP biomarker measurements from skin, TAP biomarker measurements were performed on three different skin areas from healthy volunteers (N = 10) on five consecutive days. For this, FibroTx TAP containing anti-IL-1α and -IL-1RA capturing antibody micro-arrays were incubated on normal appearing skin on cheek (face), collar bone (neck) and on the inside of the lower arm (lower forearm) of healthy volunteers for 20 minutes. FibroTx TAP capture antibody micro-arrays were collected after incubation on skin and stored at 4°C until further analysis. This procedure was repeated the four following days, at the same positions on the different skin areas, approximately at the same time of day. Captured IL-1α and IL-1RA were visualised using anti-IL-1α and -IL-1RA detection antibodies in spot-ELISA, and quantitatively analyzed, as described. As shown in Figure 6, IL-1α and IL-1RA could be efficiently detected on all three different skin areas using TAP. Notably, whereas IL-1α is found in the neck region and on the inside of the lower arm in higher amounts than IL-1RA, the reverse pattern is observed on facial skin (see Figure 6A). There was little day-to-day variation in amounts of IL-1α and IL-1RA on five consecutive days, with average CV values of 21.1% and 18.4%, respectively (see Figure 6B).

### Benchmarking of TAP protein measurements from skin with protein measurements from skin lavage

**Figure 7:**
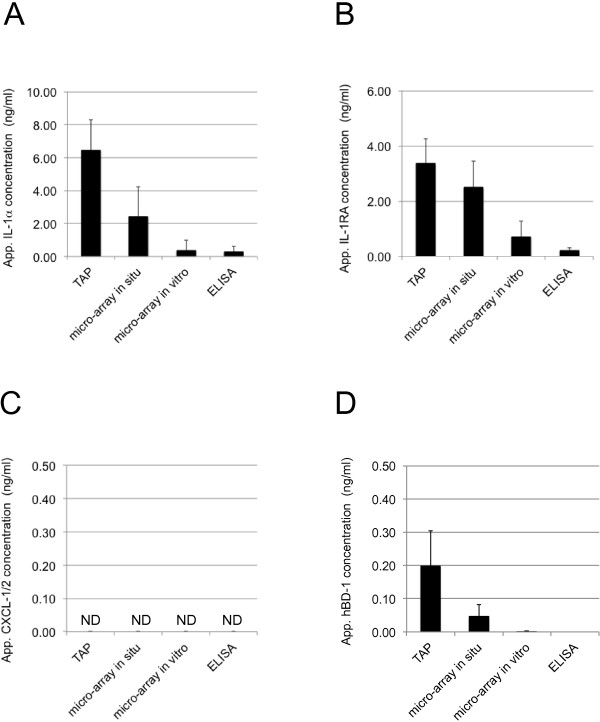
**Measurements of IL-1** **α, IL-1RA, CXCL-1/2 and hBD-1 from healthy skin using TAP and from skin lavage techniques.** TAP measurements of IL-1α, IL-1RA, CXCL-1/2 and hBD-1 from healthy skin were analyzed and compared with three different methods using skin lavage. See text for details. Signal intensities for the different methods were compared to signals obtained using fixed concentrations of recombinant IL-1α, IL-1RA, CXCL-1/2 and hBD-1 captured in solution. In the graphs, it is clearly visible that TAP is the most sensitive method for the detection of IL-1α (panel **A**), IL-1RA (panel **B**) and hBD-1 (panel **D**) on skin. CXCL-1/2 (panel **C**) could not be detected by any of the methods. Each data point in the graphs represents the results of two spots analyzed on 20 TAP’s (N = 10). Y-axis: Apparent concentration of IL-1α, IL-1RA, CXCL-1/2 or hBD-1 on skin in ng/ml. X-axis: Incubation time on skin in minutes. Error bars on graph present standard deviation of analyzed data points (N = 10).

To benchmark the efficacy of TAP protein measurements from skin, TAP protein measurements were performed in parallel using skin lavage. For this, TAP containing micro-arrays with anti-IL-1α, -IL-1RA, -CXCL-1/2 and -hBD-1 capturing antibodies were applied in two-fold to normal skin on the lower arm of healthy volunteers (N = 10). Captured IL-1α, IL-1RA, CXCL-1/2 and hBD-1 were subsequently visualised using anti-IL-1α, -IL-1RA, -CXCL-1/2 and -hBD-1 detection antibodies, in spot-ELISA, as described. Three different formats of protein detection using skin lavage were applied. In the first method, skin lavage was performed essentially as previously described by Portugal-Cohen [7, 36, 37]. In short, silicone rings were fixed to skin that served as reservoirs for PBS to extract proteins from skin. Subsequently, presence of IL-1α, IL-1RA, CXCL-1/2 and hBD-1 in skin lavage was qualitatively and quantitatively analyzed using sandwich ELISA. In the second method, skin lavage was applied to TAP micro-arrays *in vitro*, and captured IL-1α, IL-1RA, CXCL-1/2 and hBD-1 were subsequently analyzed similar to the method used for quantitative analyses of proteins captured directly from skin using TAP. In the third method, TAP micro-arrays were incubated in skin lavage on the patient *in situ* and captured IL-1α, IL-1RA, CXCL-1/2 and hBD-1 was subsequently analyzed similar to the method used for quantitative analyses of proteins captured directly from skin using TAP. All four methods were capable of detecting IL-1α, IL-1RA and hBD-1 from skin, with the exception of the ELISA method used for hBD-1 (see Figure 7, panels A, B and D). In contrast, none of the methods were capable of detecting CXCL-1/2 (see Figure 7, panel C). Clear differences were observed in the efficacy of protein measurements from the skin. TAP was by far the most sensitive method used, yielding apparent concentration values 20- (IL-1α) and 15- (IL-1RA) fold in excess of the values found using a combination of skin lavage and ELISA. Using TAP capture antibody micro-arrays for the analyses of IL-1α, IL-1RA, CXCL-1/2 and hBD-1 from skin lavage yielded only marginal improvements in detection efficacy over ELISA. Since TAP uses the same micro-arrays, this suggests that the greater efficacy of TAP may be related, at least in part, to differences in the concentration of skin proteins in skin lavage and in the fluid on the interface between micro-array and skin in the TAP procedure. Then again, applying the capture antibody micro-arrays in skin lavage *in situ* proved approximately 8- (IL-1α) and 3.5- (IL-1RA) times more efficient than applying the same skin lavage to capture antibody micro-arrays *in vitro*, suggesting that capturing proteins directly from skin, as performed during the TAP procedure and during the capturing proteins using micro-arrays *in situ*, is far more efficient than capturing of skin proteins from skin lavage *in vitro*.

## Discussion

Skin biomarker measurements have enormous value for detailed assessment of skin conditions, both for clinical applications and in skin care. Biomarkers secreted on skin can be used to assess the condition of skin at a given time-point, but may also be used to assess how a given skin condition may progress in time, or how skin may react to treatments. Skin biomarker measurements are usually performed via invasive methods that are unpleasant for patients and that require specialised laboratories for analyses. In recent years, methods have been developed for the analyses of skin biomarkers by non-invasive methods, based on the extraction of biomarkers from the surface of skin. Using skin-lavage to extract biomarkers from the surface of skin, Portugal-Cohen *et al*. have shown clear differences in expression levels of skin hydrophilic biomarkers, including cytokines (IL-1α, TNF-α and IL-6) and antioxidants (uric acid, total antioxidant scavenging capacity), between lesional and non-lesional skin in patients with atopic dermatitis and psoriasis [7, 36]. Similarly, Portugal-Cohen *et al*. found significant elevations in the expression of IL-10, TNF-α and uric acid on skin of hemodialysis patients with chronic renal failure, in comparison with healthy individuals, suggesting that skin biomarker analyses may be useful for monitoring patients with a variety of diseases with dysfunctional skin manifestations, as well [37]. Another method involves analyses of analytes from dead skin cells obtained from skin by tape stripping methods, e.g. using D-Squame tape. Using tape-stripping in combination with mass spectrometry analyses, Emson *et al*. have recently shown that they could detect marked quantitative differences in keratinocyte turn-over between lesional and non-lesional skin on psoriasis vulgaris patients [44].

TAP was designed with two goals in mind. The first goal was to develop a highly versatile and sensitive tool for accurate multi-analyte biomarker measurements from the surface of skin. The second goal was to ensure that TAP was easy to use with a minimal burden for patients. To achieve the first goal, the core of TAP was designed as a capture antibody micro-array. The capture antibody micro-arrays allow multi-analyte capturing of biomarkers directly from skin, using minimal amount of fluid between TAP and skin, ensuring much higher concentrations of biomarkers during biomarker capturing than skin lavage-based methods, or during the analyses of individual biomarkers from tape-stripping. In our benchmark studies we found that TAP was far more sensitive than antibody based methods using skin lavage techniques in our assays, which in part could be attributed to differences in the amount of fluid used for the biomarker capturing process. However, we also observed that applying capture antibody micro-arrays *in situ*, i.e. during skin lavage on skin, was more sensitive than capturing biomarkers from the same skin lavage *in vitro*. This indicates that capturing of biomarkers directly from skin surface may have other advantages, as well. At present time it is unclear what causes this effect, although one may speculate that analytes captured directly from skin may somehow be protected from degradation by capturing antibodies, which is not the case when analytes become dissolved during skin-lavage.

To provide TAP with the highest sensitivity, we optimized the printing process of the capture micro-arrays. Printing buffers can highly impact the printing of proteins on nitrocellulose, as well as the specifications of nitrocellulose itself [45, 46]. We found that PBS supplemented with 20% glycerol yielded the highest sensitivity. Since the same amounts of IgG were used for optimisation of printing, we conclude that the benefit of PBS + 20% glycerol is that it yields the most stable interaction between IgG and nitrocellulose during the biomarker binding process. Although we did not find any remarkable differences between different nitrocellulose materials used, from different suppliers, and with different porosities, we observed that Whatmann Protran BA85 (0.45 μm porosity) yielded the least variability between printed spots, and hence this material was chosen for TAP production.

In our assays to find the optimal conditions for skin biomarker measurements, using IL-1α, IL-1RA, CXCL-1/2 and hBD-1 as model analytes, we found that the amount of capture antibody printed on the capture antibody micro-array was the most important factor that determined both sensitivity and dynamic range of measuring analytes *in vitro*. The higher the amount of capture antibodies used, the highest the signal strength, sensitivity and dynamic range of measurements achieved in our experiments. The combination of amount of detection antibody used in our biomarker analyses, and concentration of amplification reagents used, impacted predominantly the curve fit of the concentration range, and thus the accuracy of measurements that can be achieved. Optimal settings, which changed somewhat between analytes measured, yielded R^2^ values of over 0.99, thus indicating that highly accurate biomarker measurements with TAP can be achieved.

To achieve the second goal, convenience of use, we designed TAP as an adhesive bandage. The bandage allows easy fixture of the TAP capture antibody micro-array to skin. This, and the fact that TAP uses a sponge layer as an expandable fluid reservoir that ensures close contact between the micro-array and skin, makes TAP insensitive to gravity issues e.g. such as observed using skin-lavage techniques. In addition, the relatively small size of the TAP micro-array allows measurements of small skin lesions, e.g. as observed in certain forms of psoriasis or bacterial infections, e.g. from tick bites. The methods used for TAP biomarker analyses have been based in large on well-established techniques. In essence, TAP biomarker analyses are based on sandwich ELISA, which is a standard and cost-effective technique for qualitative and quantitative protein analyses established in laboratories all over the world. The design of the micro-array allows for easy adaptation to different sets of capture and detection antibodies used, and thus specific TAP’s for specific purposes can be designed with relative ease. Using TAP, we could detect IL-1α, IL-1RA and hBD-1 from normal skin of healthy volunteers, but not CXCL-1/2 due to its low expression on normal skin. Measuring IL-1α and IL-1RA on the skin of healthy volunteers on five consecutive days, we could show that TAP biomarker levels fluctuated with a CV of around 20%. Since the CV values of TAP measurements under ideal conditions are around 5%, we conclude that there is a low day-to-day variation in biomarker presence on skin. From these measurements it became apparent that the ratio between IL-1a and IL-1RA is different on skin in the lower inner arm and neck regions, in comparison with facial skin, which is very much in alignment with tape-stripping studies performed by Hirao et al. showing that IL-RA is much higher expressed the stratum corneum of sun-exposed skin areas (e.g. the face) than in areas (e.g. inner forearm) not exposed to sun-light [47]. These biomarkers were chosen because they have been described as regulated proteins in different skin conditions, such as atopic dermatitis and psoriasis. Currently, studies are ongoing with TAP to study the expression of various biomarkers in different skin conditions, including psoriasis, and we can see clear differences between skin affected and skin not affected by disease (manuscript in preparation). Using TAP on skin, no adverse effects, such as redness or itching were observed, and thus we can conclude that TAP is a highly convenient and versatile new diagnostic tool for non-invasive biomarker measurements from skin that is safe to use.

## Conclusions

With TAP, we could capture and measure a selection of regulatory proteins expressed in the skin, IL-1α, IL-1RA and hBD-1, from the surface of normal skin of healthy volunteers, thus presenting TAP as a practical and highly sensitive skin-diagnostic tool for measuring protein-based biomarkers from skin with minimal effort for operators, as well as with minimal burden for persons being analyzed. No adverse effects, such as redness or itching were observed, and thus we conclude that TAP is a highly convenient and versatile new diagnostic tool for non-invasive biomarker measurements from skin that is safe to use.

## Methods

### Antibodies

Human GRO-ß (CXCL-2) ELISA Development Kit (Cat. No: 900-K120, PeproTech), Human IL-1α ELISA Development Kit (Cat. No: 900-K11, PeproTech), Human hBD-1 ELISA Development Kit (Cat. No: 900-K202, PeproTech), Human IL-1RA ELISA Development Kit (Cat. No: 900-K474, PeproTech), Human IgG (Lab AS, Estonia), Goat anti-human IgG Lab AS, Estonia).

### Human IgG printing on nitrocellulose

Human IgG was diluted in PBS (pH = 7.4) with 10% or 20% (v/v) glycerol (Scharlau Chemie), supplemented with either 0.1% or 0.05% Triton X-100 (Scharlau Chemie), 0.5% Tween-20 (AppliChem), 1% ethanol or no supplement to concentrations of 2.5, 5, 10, 20 and 40 μg/ml. Droplets (30 nl) containing 0.075, 0.15, 0.3, 0.6 and 1.2 ng per spot of human IgG were deposited on Whatman Protran BA 85 nitrocellulose membrane strips, with porosities of either 0.1, 0.2 or 0.45 μm, or Amersham Hybond C-Extra, with 0.45 μm porosity, using a non-contact dispenser printer (AD3400, BioDot). Each protein amount was printed in 5 replicates. To avoid any unspecific protein binding, nitrocellulose strips were blocked with blocking buffer (PBS + 5% (w/v) BSA (PAA Laboratories GmhH) for one hour at room temperature. Strips were dried at room temperature after washing in PBS and milli-Q water and stored at 4°C.

For FibroTx TAP development pre-washed and dried nitrocellulose strips of Whatman Protran BA 85 (0.45 μm porosity) containing 0.75, 1.5 and 2.25 ng /spot anti-IL-1α, -IL-1RA, -hBD-1 and -CXCL-2 antibodies dissolved in PBS (pH = 7.4) and 20% (v/v) glycerol were generated essentially as described.

### Visualisation of printed human IgG spots using spot-ELISA

Nitrocellulose strips with printed human IgG spots were incubated with 1 μg/ml HRP-conjugated goat anti-human IgG in PBS + 0.05% (v/v) Tween-20 + 1% (w/v) BSA for 45 minutes at room temperature. After washing with PBS + 1% (w/v) BSA + 0.1% (v/v) Tween-20, human IgG spots were visualised using 20% Substrate-Chromogen solution diluted in Substrate Buffer Concentrate (Dako, K-1500). The reaction was stopped with milli-Q water.

### Visualisation of captured proteins using spot-ELISA

For the standard curves, recombinant IL-1α, IL-1RA, hBD-1 and CXCL-2 were diluted in PBS, 0.05% (v/v) Tween-20, 1% (w/v) BSA and incubated for 30 min at 33°C to mimic skin temperature. Unbound proteins were washed from membrane with wash buffer containing PBS, 1% (w/v) BSA, 0.1% (v/v) Tween-20. Biotinylated secondary antibody were added to membrane strips in two concentrations and incubated for 45 min at room temperature. For signal amplification Catalysed Signal Amplification (CSA) System (Dako, K-1500) was used. Streptavidin-Biotin Complex, Amplification Reagent and Streptavidin-HRP were diluted in PBS, 0.05% (v/v) Tween-20 up to 10% or 20%. For signal visualisation Substrate-Chromogen solution diluted in Substrate Buffer Concentrate was used. The reaction was stopped with milli-Q water.

### Biomarker measurements from skin

Ethical approval for the studies was covered by the Tallinn Medical Research Ethical Committee (Decision No. 2551). No visual signs of a reaction on skin towards the FibroTx TAP capture antibody micro-array system, such as itching, redness or any other discomfort was observed.

### Skin lavage

A four-ring silicon device (Greiner Bio-One) was placed on the inside of the lower arm of healthy volunteers (N = 10) and fixed with a tubular net bandage (Kaigert). All rings were filled with 900 μl PBS (pH = 7.4) and incubated on skin for 30 minutes. For the capturing of biomarkers directly from skin, TAP capture antibody micro-arrays were placed into two rings during skin lavage. After incubation, lavage solution where no TAPs were present was collected into sterile 15 ml tubes (Greiner Bio-One). Micro-arrays incubated in skin lavage were collected into 1.5 ml tubes (Eppendorf) and stored at 4°C until further analysis.

### Enzyme-linked immunosorbent assay (ELISA)

The levels of hBD-1, CXCL-1/2, IL-1α and IL-1RA secreted into skin surface were measured using human hBD-1, Gro-β (which identifies both CXCL-1 (Gro-α) and -2 (Gro-β) in our assays), IL-1α and IL-1RA ELISA Development Kits. In short, skin lavage liquid was centrifuged at 13. 000 rpm for 5 minutes and 50 μl /well of fluid was analyzed in high binding ELISA plates (Greiner BioOne), at room temperature, according to the manufacturer's instructions. Signal optical density was measured using a Spectramax 340 PC photospectrometer (Molecular Devices) 450 nm.

### TAP biomarker measurements on skin

Biomarker measurements from skin were performed using TAP capture antibody micro-arrays. Each micro-array, printed on nitrocellulose membrane, contained two spots of biomarker capturing antibody: 0.75 ng and 1.5 ng of IL-1α, 2.25 ng hBD-1, 2.25 ng CXCL-2, 2.25 ng IL-1RA per spot, additionally, each micro-array contained a negative control (PBS-with 20% (v/v) glycerol) and positive control (0.03 ng biotinylated anti- hBD-1).

In order to assess the amounts of IL-1α, IL-1RA, CXCL-1/2 and hBD-1 on skin, TAP capture antibody micro-arrays coated with anti-IL-1α, anti-IL-1RA, anti-CXCL-1/2 and anti-hBD-1 were applied in duplicates to the inside of the lower arm of healthy volunteers (N = 10), both men and women. FibroTx TAP capture antibody micro-arrays were wetted on skin with 150 μl PBS (pH = 7.4) and incubated for 30 minutes. Following incubation, TAP capture antibody micro-arrays were collected from the skin and stored at 4°C until further analysis. Captured IL-1α, IL-1RA, CXCL-1/2 and hBD-1 were visualised using spot-ELISA.

### Spot-ELISA

48-well plates (Greiner BioOne) were blocked with 1% BSA (w/v) in PBS (pH = 7.4). Subsequently, wells were washed with milli-Q water and dried. For further processing TAP capture antibody micro-arrays were placed into blocked 48-well plates and wetted with PBS.

To create standard curves, capture antibody micro-arrays were incubated for 30 minutes at 33°C with a mixture of hBD-1, CXCL-2, IL-1α and IL-1RA recombinant proteins diluted in PBS + 0.05% (v/v) Tween-20 + 1% (w/v) BSA. For analyses of biomarkers from skin lavage, TAP micro-arrays were incubated with 150 μl/well of lavage solution for 30 min at 33°C. The capture antibody micro-arrays were washed subsequently with PBS + 0.1% (v/v) Tween-20 + 1% (w/v) BSA before further processing, similar to FibroTx TAP capture antibody micro-arrays collected from skin and from lavage solution.

Subsequently the micro-arrays were washed with wash buffer and incubated with specific biotinylated detection antibodies mix containing anti-hBD-1 (333.33 ng/ml), anti-CXCL-1/2 (166.67 ng/ml), anti-IL-1α (142.86 ng/ml) and anti-IL-1RA (166.67 ng/ml), for 45 min at room temperature. For micro-array signal enhancement, 10% CSA System components were used as described. Signal was visualised using 20% Substrate-Chromogen solution diluted in Substrate Buffer Concentrate. Reaction was stopped with milli-Q water.

Signals of captured IL-1α, IL-1RA, CXCL-1/2 and hBD-1 were quantified by comparing the signals of these proteins captured from skin of healthy volunteers using FibroTx TAP capture antibody micro-array and lavage method with signals from FibroTx TAP capture antibodies micro-arrays incubated with fixed amounts of recombinant IL-1α, IL-1RA, CXCL-1/2 and hBD-1.

### TAP biomarker measurements on skin for biomarker variation determination in time

Biomarker measurements from skin were performed using TAP capture antibody micro-arrays containing three spots of biomarker capturing antibody: 0.3 ng of IL-1α and 1.125 ng IL-1RA per spot, additionally each micro-array contained a negative control (PBS-with 20% (v/v) glycerol) and positive control (0.03 ng biotinylated anti- hBD-1). TAP capture antibody micro-arrays coated with anti-IL-1α, anti-IL-1RA were applied in duplicates to the face area, collar bone and inside of the lower arm of healthy volunteers (N = 10), including both men and women. FibroTx TAP capture antibody micro-arrays were incubated on skin for 20 minutes. Following incubation, TAP capture antibody micro-arrays were collected from the skin and stored at 4°C until further analysis. Captured IL-1α and IL-1RA, were visualised using spot-ELISA. Signals of captured IL-1α and IL-1RA were quantified by comparing the signals of these proteins captured from skin of healthy volunteers using FibroTx TAP capture antibody micro-array with signals from FibroTx TAP capture antibodies micro-arrays incubated with fixed amounts of recombinant IL-1α and IL-1RA.

### Image analyses

Processed TAP capture antibody micro-arrays and nitrocellulose strips were scanned with a flat-bad scanner (Canon) at 1200 dpi resolution, using a 0–255 grey-scale, and analyzed with Adobe Photoshop Elements software. Processed TAP capture antibody micro-arrays for biomarker variation determination in time were scanned with FibroTx TAP scanner, using a RGB scale. On both cases biomarker signals were corrected for background staining.

## Abbreviations

CV: Coefficient of variation
hBD-1: Human β-defensin-1
hBD-2: Human β-defensin-2
IL-1α: Interleukin-1α
IL-1β: Interleukin-1β
IL-1RA: Interleukin-1 receptor antagonist
CCP: Cyclic-citrulinated peptide
CXCL-1: Chemokine (C-X-C motif) ligand 1
CXCL-2: Chemokine (C-X-C motif) ligand 2
IL-6: Interleukin-6
IL-10: Interleukin-10
TNF-α: Tumor necrosis factor α
IFN-γ: Interferon γ
IgG: Immunoglobulin G
ng: Nanogram
μm: Micrometer
μl: Microliter
VEGF: Vascular endothelial growth factor
PCT: Patent cooperation treaty
pg: Picogram
SD: Standard deviation
TAP: Transdermal analyses patch
ELISA: Enzyme-linked immunoso rbent assay
PASI: Psoriasis area severity index
RGB: Red-green-blue color model
SCORAD Index: SCORing atopic dermatitis index.
